# Zika Virus Testing and Outcomes during Pregnancy, Florida, USA, 2016

**DOI:** 10.3201/eid2401.170979

**Published:** 2018-01

**Authors:** Colette Shiu, Rebecca Starker, Jaclyn Kwal, Michelle Bartlett, Anise Crane, Samantha Greissman, Naiomi Gunaratne, Meghan Lardy, Michelle Picon, Patricia Rodriguez, Ivan Gonzalez, Christine L. Curry

**Affiliations:** University of Miami Miller School of Medicine, Miami, Florida, USA (C. Shiu, R. Starker, J. Kwal, M. Bartlett, A. Crane, S. Greissman, N. Gunaratne, M. Lardy, M. Picon, P. Rodriguez, I. Gonzalez, C.L. Curry);; Jackson Memorial Hospital, Miami (P. Rodriguez, I. Gonzalez, C.L. Curry)

**Keywords:** Zika virus, pregnancy, public health, viruses, Florida, vector-borne infections, United States, congenital Zika syndrome, microcephaly, screening

## Abstract

Zika virus infection during pregnancy can lead to congenital Zika syndrome. Implementation of screening programs and interpretation of test results can be particularly challenging during ongoing local mosquitoborne transmission. We conducted a retrospective chart review of 2,327 pregnant women screened for Zika virus in Miami–Dade County, Florida, USA, during 2016. Of these, 86 had laboratory evidence of Zika virus infection; we describe 2 infants with probable congenital Zika syndrome. Delays in receipt of laboratory test results (median 42 days) occurred during the first month of local transmission. Odds of screening positive for Zika virus were higher for women without health insurance or who did not speak English. Our findings indicate the increase in screening for Zika virus can overwhelm hospital and public health systems, resulting in delayed receipt of results of screening and confirmatory tests and the potential to miss cases or delay diagnoses.

Zika virus infection during pregnancy can lead to congenital Zika syndrome (*1*), of which microcephaly is one of many possible malformations (*2*). Clinicians can recommend laboratory screening for Zika virus during pregnancy, even in the absence of symptoms of infection, if concern exists about exposure of the pregnant woman or her sex partner(s) because of travel to or residence in an area of ongoing Zika virus transmission (*3*). If a pregnant woman or a partner with whom she has had unprotected sex experiences symptoms, testing is warranted (*4*).

To appropriately evaluate infants born with congenital malformations, pediatricians must be aware of maternal risk for infection during pregnancy. Implementation of screening guidelines and testing for Zika virus is complicated by interpretation of test results and the need for confirmatory testing, which can delay diagnosis during a time-limited situation, such as pregnancy (*5**,**6*). To assess clinical outcomes and challenges associated with Zika virus screening and testing, we analyzed data from 2 tertiary care centers that provided care to women with travel-associated and local Zika virus infection during pregnancy.

## Methods

We retrospectively reviewed charts of all 2,327 pregnant women who were tested for Zika virus during January 1, 2016–December 31, 2016, at 2 tertiary care hospitals in Miami–Dade County, Florida, USA: University of Miami Miller School of Medicine and Jackson Memorial Hospital. After institutional review board approval, we manually extracted data from the electronic medical record. Demographic and laboratory data recorded for pregnant women tested for Zika virus consisted of age, patient-reported ethnic group, language preference, insurance status, screening test date, result receipt date, test result, number of tests performed per patient, and timing of test and result relative to delivery date. We also collected delivery outcomes and laboratory and imaging results for infants of these women.

Testing for Zika virus during pregnancy followed current guidelines from the Centers for Disease Control and Prevention (CDC; Atlanta, GA, USA). The Florida Department of Health (FLDOH) and its contracting laboratory, LabCorp (Burlington, NC, USA), modified the CDC guidelines by using Zika virus real-time reverse transcription PCR (rRT-PCR) and IgM to screen blood (IgM and rRT-PCR) and urine (rRT-PCR) samples simultaneously, regardless of symptoms or time from potential Zika virus exposure. All samples were collected at a regular obstetrics visit and sent in daily batches to the local FLDOH laboratory, which triaged them.

Before local transmission began in July 2016, laboratory testing of pregnant women was based on Zika virus exposure history (i.e., travel or sexual contact). After documented local transmission, we routinely offered laboratory screening for Zika virus to all pregnant women. If a woman had not been tested for Zika virus during her pregnancy, she was offered testing on arrival to labor and delivery. When a woman had any laboratory evidence of Zika virus infection during her pregnancy or when a congenital malformation in her neonate prompted evaluation for congenital Zika syndrome, urine, serum, or other relevant samples were sent from the neonatology inpatient service directly to the FLDOH, which tested samples directly or forwarded them for testing to LabCorp. The ordering clinician did not determine which entity tested the samples or the type of testing done.

If rRT-PCR or IgM testing yielded positive results at LabCorp or FLDOH, specimens were forwarded to CDC for plaque-reduction neutralization testing (PRNT). Women and infants who were eligible for the US Zika Pregnancy Registry were reported by FLDOH (*7*). All results were faxed to the tertiary care hospital where the specimens had been drawn.

We calculated test result delay on the basis of sample collection date and the date the hospital received the results. Women were triaged to consultation with the high-risk obstetrics team and the pediatric infectious disease team if Zika virus RNA was detected by rRT-PCR or if Zika virus IgM was detected by IgM antibody-capture ELISA (MAC-ELISA) in maternal serum. This change in care was done as part of clinical management. Women with rRT-PCR–positive serum or urine were considered to have acute Zika virus infection. Women with positive Zika virus IgM were presumed to have Zika virus infection until PRNT results were returned, after which we followed the CDC guidelines (*6*). Women with negative serum and urine rRT-PCR results and any nonnegative Zika virus IgM and with a PRNT titer for Zika virus <10 were considered to have no evidence of Zika virus. Results for which the rRT-PCR was negative, the IgM was positive, and the PRNT was <10 were considered false-positive (*8*). Results with nonnegative Zika virus IgM, Zika virus PRNT >10, and dengue virus PRNT <10 were considered to be infected with Zika virus, with timing of infection undetermined. Results with Zika virus and dengue PRNTs >10 were considered to indicate flavivirus infection, specific virus not determined.

Because missing Zika virus infection during pregnancy has consequences for the woman, her infant, and pediatric care, infected patients were managed clinically as having any laboratory evidence of Zika virus infection in pregnancy. Because some patients did not receive PRNT results during the study period, we used CDC guidelines for areas (Puerto Rico) where PRNT was not recommended and relied on the IgM results (*9*).

For women with laboratory evidence of Zika virus infection during pregnancy, we collected data on gravidity, parity, possible Zika virus symptoms, antenatal ultrasonography, length of time patient was positive for Zika virus by rRT-PCR, follow-up status, and pregnancy outcome. We calculated length of time the woman was positive for Zika virus by rRT-PCR on the basis of the date of the first and last positive rRT-PCR result. FLDOH recommended weekly rRT-PCR testing until urine and serum rRT-PCR results were negative, which provided multiple data points. Women or infants were classified as lost to follow-up after 2 missed clinic visits, 3 nonresponses to phone calls, and no response to a certified letter, as was part of our routine clinical protocol.

We also recorded outcomes of infants born to mothers with laboratory evidence of Zika virus infection during pregnancy. We reviewed infant medical records for results of Zika virus testing, neurologic imaging, auditory and ocular testing, head circumference at birth, and follow-up status.

We performed statistical analyses using SAS University Edition (SAS Institute, Inc., Cary, NC, USA). Descriptive statistics were presented as means ± SDs or medians according to the statistical distribution of continuous data and as number of patients and percentages for categorical parameters. To examine the association between insurance status, primary language, race/ethnicity, and clinical result status among the pregnant women, we used χ^2^ tests and reported p values for each test. We generated logistic regression models to estimate the effects of insurance status, primary language, and race/ethnicity on women’s clinically positive result status. Results were reported as odds ratios (ORs) with 95% CIs. We excluded from the χ^2^ and logistic regression analyses women whose test results were still pending. We also excluded women in the Native American and other race/ethnicity categories from the χ^2^ test and logistic regression model between race/ethnicity and result status because no women in these groups tested positive for Zika virus. We also excluded from individual statistical analysis patients with missing data.

## Results

**Figure 1:**
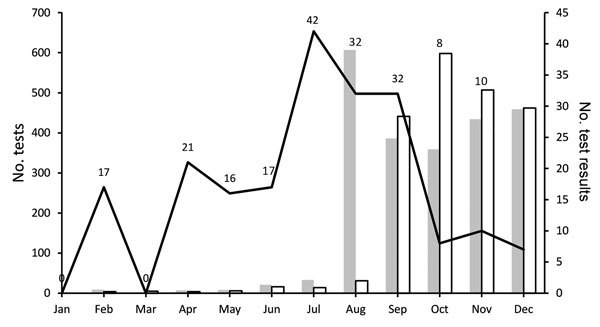
Zika virus screening tests, results, and length of result delay, by month, Miami–Dade County, Florida, USA, 2016. Numbers above line indicate median length of delay (in days) for test conducted in that month.

**Figure 2:**
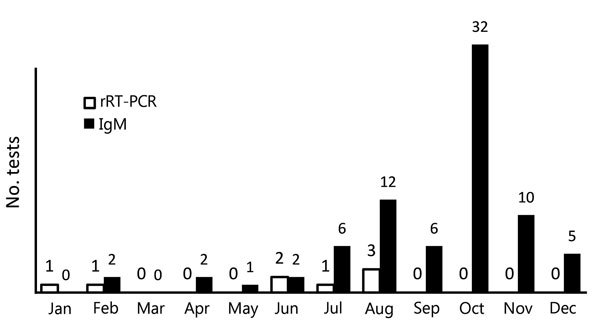
Positive Zika virus test results among pregnant women, by month and testing type, Miami–Dade County, Florida, USA, 2016. rRT-PCR, real-time reverse transcription PCR.

During 2016, a total of 2,327 pregnant women were tested for Zika virus (Table 1). Based on the ≈32,000 births reported in Miami-Dade County during 2015, the most recent year for which data are available, we estimated that our analysis represents ≈7% of births in the county (*10*). During August 2016, the month when the highest number of women (607) were screened, results were returned within that same month for only 2.6% (Figure 1). The highest number of test results (598) were returned in October, which was also the month when the greatest number of tests returned were positive for Zika virus (Figure 2). Each woman was screened for Zika virus an average of 1.12 times during her pregnancy. For 646 (27.8%) women, Zika virus testing was first performed at delivery (Table 2). Including women tested at delivery, patients with delays in result receipt and women tested in the third trimester, 37% of results were received after delivery (Table 2).

**Table 1:** Demographic characteristics of 2,327 pregnant women tested for Zika virus, Miami–Dade County, Florida, USA, 2016

Characteristic	Result
Age, y, mean ± SD	28.9 ± 6.09
Race/ethnicity,* no. (%)	
Non-Hispanic white	262 (11.3)
Non-Hispanic black	741 (31.8)
Hispanic	996 (42.8)
Haitian	265 (11.4)
Asian/Pacific Islander	42 (1.8)
Native American	2 (0.1)
Other	19 (0.8)
Insurance, no. (%)	
Public	1,535 (66.0)
Private	350 (15.0)
Not insured	442 (19.0)
Primary language, no. (%)	
English	1,472 (63.3)
Spanish	609 (26.2)
Haitian Creole	214 (9.2)
Other	32 (1.4)

*Race/ethnicity listed as “ethnic group” and was patient self-identified.

**Table 2:** Laboratory test results of 2,327 pregnant women for Zika virus, Miami–Dade County, Florida, USA, 2016

Laboratory characteristic	Result
Test results, no. (%)	
IgM positive	102 (4.4)
rRT-PCR–positive	8 (0.3)
Negative	1,999 (85.9)
No. tests per patient, mean ± SD	
Overall	1.12 ± 0.44
Positive	1.76 ± 1.51
Negative	1.10 ± 0.33
Timing of test, no. (%)	
At delivery	646 (27.8)
Before delivery	1,681 (72.2)
Receipt of result, no. (%)	
Before delivery	1,312 (56.4)
After delivery	860 (37.0)
Undetermined	155 (6.7)

**Figure 3:**
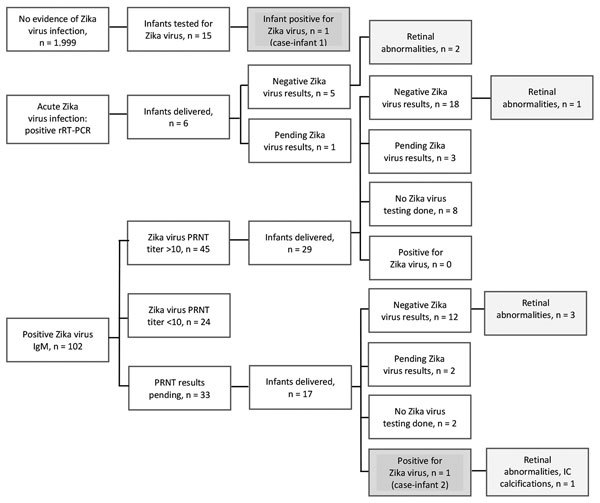
Maternal and infant Zika virus test results and outcomes, Miami–Dade County, Florida, USA, 2016. IC, intracranial; PRNT, plaque-reduction neutralization testing; rRT-PCR, real-time reverse transcription PCR.

Of the 2,327 women screened, 1,999 (85.9%) had no laboratory evidence of Zika virus during pregnancy (Table 2). Eight (0.34%) women had evidence of acute Zika virus infection by positive rRT-PCR. For 102 (4.4%) women, IgM results were presumptive for recent Zika virus infection (Table 2). Of the 69 for whom we received PRNT results, 24 (34%) had results <10 for Zika virus, which met CDC criteria for no evidence of Zika virus infection, so the initial tests were considered false-positive (*8*). For 10 (41%) women, PRNT results for dengue were >10. For 33 women with presumptive recent Zika virus infection, PRNT results were not available during the study period, and these women were managed as presumptively positive. The remaining 45 women had PRNT results >10 for Zika virus; for 40 (88%) of these, PRNT results were >10 for dengue virus (Figure 3).

Of women with acute Zika virus infection, rRT-PCR documented prolonged Zika virus in 6 women. Two of these had 1 positive rRT-PCR time point; for the rest, time from first to last positive rRT-PCR result were 13, 55, 48, 19, 25, and 13 days. For the remainder of our analysis, pregnant women with evidence acute Zika virus infection, women with evidence of Zika virus (timing of infection undetermined), and women with presumptive Zika virus (IgM positive, PRNT results not available) were analyzed together as having any laboratory evidence of Zika virus infection during pregnancy because their clinical management during pregnancy and the subsequent testing of the infants was the same.

Of the 86 pregnant women with laboratory evidence of Zika virus infection, 59 (68.6%) had >2 previous pregnancies (Table 3). Fifty-three (61.6%) of the 86 women were asymptomatic; 14 (16.2%) had documented symptoms suspicious for Zika virus infection. Local acquisition of Zika virus was suspected for 40 (46.5%) women (no documented travel for patient or partner during pregnancy); 26 (30.2%) women were thought to have travel-associated infection. By the end of 2016, 44 (51.1%) pregnant women with laboratory evidence of Zika virus had delivered their infants at term, and 8 (9.3%) women had preterm deliveries (Table 3). Twenty-three (26.7%) women were lost to follow-up for prenatal care.

**Table 3:** Characteristics of 86 women with laboratory evidence of Zika virus infection during pregnancy, Miami–Dade County, Florida, USA, 2016

Characteristic	No. (%)
Gravidity	
1	27 (31.0)
>2	59 (68.6)
Parity	
0	8 (9.3)
1	39 (45.3)
>2	39 (45.3)
Reason tested	
Asymptomatic	53 (61.6)
Symptomatic	14 (16.2)
Not determined	19 (22.1)
Pregnancy outcome	
Preterm delivery	8 (9.3)
Term delivery	44 (51.1)
Still pregnant	34 (39.5)
Location of virus acquisition	
Local	40 (46.5)
During travel	26 (30.2)
Both	15 (17.4)
Undetermined	5 (5.8)
Follow-up	
Lost to follow-up	23 (26.7)
Continued care	63 (73.3)

We assessed outcomes for 52 infants of women with laboratory evidence of Zika virus during pregnancy and 1 infant with laboratory-confirmed Zika virus for whom maternal rRT-PCR and IgM were negative for Zika virus (Table 4). Two infants had probable congenital Zika virus infection (Table 5); because 1 (case-infant 1) was born to a pregnant woman without laboratory evidence of Zika virus infection, we excluded this infant from our calculations, except where indicated. The remaining infants with ocular or imaging abnormalities were negative by rRT-PCR and IgM and therefore were considered negative for congenital Zika virus infection. However, neonatal testing might have been performed after viral RNA and IgM had cleared (*11*).

**Table 4:** Outcomes and characteristics of neonates from 86 pregnant women who had laboratory evidence of Zika virus during pregnancy, Miami–Dade County, Florida, USA, 2016

Characteristic	No. (%)	
Delivery status		
Delivered	53 (60.9)	
In utero	34 (39.1)	
Testing status		
Tested	43 (81.1)	
Not tested	10 (18.9)	
Test result		
Positive	2 (4.7)	
Negative	39 (90.7)	
Pending	2 (4.7)	
Follow-up		
Lost to follow-up	21 (39.6)	
Continued care	32 (60.4)	
Head circumference at birth	
Abnormal	5 (9.4)
Within normal limits	48 (90.6)
Audiology testing	
Abnormal	1 (1.9)
Normal	43 (81.1)
Not tested	9 (17.0)
Fundoscopic exam results	
Abnormal	8 (13.2)
Normal	9 (16.9)
Pending	13 (24.5)
Not tested	24 (45.3)
Cranial magnetic resonance imaging results	
Abnormal	2 (3.8)
Not tested	51 (96.2)
Cranial ultrasound at birth	
Abnormal	9 (17.0)
Normal	29 (54.7)
Not tested	15 (28.3)

**Table 5:** Clinical and laboratory characteristics of 2 infants and their mothers who had laboratory evidence of Zika virus infection, Miami–Dade County, Florida, USA, 2016*

Characteristic	Case-infant 1	Case-infant 2
Country of exposure	Haiti	Venezuela
Gestational age at time of symptoms	≈10 wk (2015 Nov)	≈12 wk (2015 Dec)
Laboratory results for Zika virus		
Mother	Serum IgM neg (April 2016)	Serum neg rRT-PCR, pos IgM (2016 Apr)
Infant	Serum/CSF neg rRT-PCR, pos IgM, pos PRNT >10 Zika virus (2016 May)	Serum/CSF/CB neg rRT-PCR, pos IgM (2016 Jun)
Antenatal ultrasound	HC <3%, BPD <3% (33.1 WGA)	HC 10%, BPD 34% (36.4 WGA)
HC at birth	30.5 cm (<1%)	34 cm (25%–50%)
Postnatal cranial imaging	Serpiginous calcifications, R; polymicrogyriccortex, BL; simplified gyral pattern (BL, L >R)	Linear calcifications, L; polymicrogyric cortex, R; atrophy cerebral peduncle, R; overall volume loss of entire brain, R >L
Ocular evaluation	Unremarkable	Hypopigmented R superior retinal lesion
Auditory evaluation	Unremarkable	Normal

*BL, bilateral ; BPD, biparietal diameter; CSF, cerebrospinal fluid; HC, head circumference; L, left; neg, negative; pos, positive; PRNT, plague-reduction neutralization test; R, right; rRT-PCR, real-time reverse transcription PCR; WGA, weeks gestational age.

None of the antenatal ultrasounds for 66 women for whom they were documented showed intracranial calcifications. For 2 infants, intracranial calcifications noted after birth were not detected before delivery (Table 4). For 5 (9.4%) infants, head circumference at birth was reported as below the third percentile; only 1 (case-infant 1; Table 5) met criteria for microcephaly. Of the 52 infants born to women with evidence of Zika virus infection, Zika virus testing was done on 43. The untested infants were discharged home before receipt of maternal positive Zika virus testing results and did not return for care. Ocular abnormalities were documented for 7 infants (including case-infant 2; Table 5). One infant also had auditory abnormalities (Table 4). Ocular defects were reported as retinal hemorrhage, abnormalities of the optic nerve, severe attenuation of normal retinal vasculature, anomalies of the optic nerve, and abnormal hyaloid artery development. If we consider the 2 infants with probable congenital Zika virus infection, then 2 (3.7%) infants were affected. If we include the additional 6 infants who had ocular/retinal abnormalities but who had negative results, then 8 (15.0%) infants were affected. Twenty-one (39.6%) infants were lost to follow-up.

A total of 65.3% of pregnant women who had laboratory evidence of Zika virus infection had public insurance, 15.8% had private insurance, and the remaining 18.9% were uninsured (Table 6). We found a significant association between insurance status and Zika virus test result (p<0.0001 by χ^2^ test). Uninsured patients had higher odds of receiving a positive Zika virus test result (OR 3.08, 95% CI 1.95–4.86) than did women with public insurance. 

**Table 6:** Association between race/ethnicity, insurance status, and language among Zika virus–positive pregnant women, Miami–Dade County, Florida, USA, 2016*

Characteristic	No. (%) women	Odds ratio (95% CI)	p value
Race/ethnicity*			0.0013
Non-Hispanic white	231 (11.2)	Reference	
Non-Hispanic black	655 (31.7)	0.60 (0.23–1.54)	
Hispanic	903 (43.7)	1.88 (0.84–4.19)	
Haitian	235 (11.4)	2.34 (0.94–5.79)	
Asian/Pacific Islander	42 (2.0)	1.60 (0.32–7.98)	
Insurance			<0.0001
Public	1,362 (65.3)	Reference	
Private	329 (15.8)	0.76 (0.36–1.64)	
Uninsured	395 (18.9)	3.08 (1.95–4.86)	
Language			0.0001
English	1,316 (63.1)	Reference	
Spanish	554 (26.6)	2.62 (1.63–4.21)	
Haitian Creole	190 (9.1)	2.91 (1.54–5.52)	
Other	26 (1.3)	1.46 (0.19–11.11)	

*The Native American and other categories were excluded because there were no clinically positive results in these racial/ethnic groups (complete prediction).

Language preference was significantly associated with Zika virus test result (p = 0.0001). Patients speaking Spanish and Haitian Creole had higher odds of receiving a positive Zika virus test result than English speakers (OR 2.62 and 2.91, respectively). Race/ethnicity also was significantly associated with Zika virus test result p = 0.0013 by χ^2^ test). However, the ORs for each race/ethnicity compared with non-Hispanic white patients were not significant (95% CIs all included 1).

## Discussion

We report on the clinical outcomes, challenges in testing, and social factors associated with screening positive for Zika virus infection during pregnancy. Of the 52 infants born to women with evidence of Zika virus infection, 2 (3.7%) had evidence of probable congenital Zika virus infection, both from first trimester Zika virus infections. Difficulty in estimating the true percentage of infants affected by Zika virus is challenging because current testing might not provide laboratory evidence of fetal Zika virus infection after delivery (*12*). Additional challenges to understanding the true incidence of congenital Zika syndrome also might be related to access to Zika virus testing. The mother of case-infant 1 did not have laboratory evidence of Zika virus during pregnancy; astute pediatric care enabled detection. The mother of case-infant 2 had Zika virus IgM without PRNT results and was presumed to have been infected during pregnancy. Broad application of laboratory testing of infants enabled case detection. Current testing modalities make attributing other abnormalities, such as retinal damage, to Zika infection during pregnancy challenging (*13*).

In the cohort we report, delays in receipt of results of Zika virus screening occurred during the first half of 2016. The longest delays occurred in before local mosquitoborne transmission began; delays decreased as the laboratories and public health agencies became accustomed to an increased number of laboratory tests. In the context of pregnancy, delays in result reporting may affect decisions about continuation and termination of pregnancy (*6**,**12**,**14**,**15*). An additional challenge to patient management is the known cross-reactivity of current IgM tests with antibodies from past infections with related flaviviruses (*16**,**17*). Forty-one percent of the women in this study who had false-positive Zika virus IgM test results had PRNT results demonstrating previous infection with dengue virus. During counseling and disclosure of test results, the ability of the patient and provider to tolerate uncertainty cannot be overstated (*18*). Concern about false-positive Zika virus test results should be balanced with concern about missing an infant exposed to Zika virus during pregnancy.

Recent clinical guidelines recommend including rRT-PCR in screening during each trimester of pregnancy to add specificity in detecting Zika virus. Zika virus IgM is detectable for longer than previously anticipated; the median time from seroconversion to IgM negative is 122 days and as long as 210 days (*19*). The longevity of the Zika virus IgM response makes determining trimester of infection, or possible preconception infection, under previous screening guidelines more difficult to interpret (*9**,**19**,**20*). FLDOH has used both IgM and rRT-PCR as part of the screening since recognizing local transmission in July2016. Even with simultaneous IgM and rRT-PCR laboratory testing, only 8 women had positive rRT-PCR results. Therefore, the value of increasing the number of cases detected by adding rRT-PCR to national guidelines is questionable, although specificity is enhanced when the rRT-PCR is positive. Conversely, including Zika virus IgM screening for pregnant women with ongoing exposure carries the risk for false-positive results as the incidence of disease decreases, but such screening should be discussed with patients as a valuable tool because current diagnostic testing options remain limited (*18**,**21*).

As part of the public health response to local transmission of Zika virus in 2016, Florida state authorities made access to Zika virus screening free for all pregnant women through FLDOH. Review of the cohort reported here suggests that removal of financial barriers to screening were important; 18.9% of the women in this study had no insurance and had increased odds of testing positive for Zika virus during pregnancy. We consider removal of financial barriers to screening as an important adjunct to provider counseling. Similarly, pregnant women in this cohort who primarily spoke Spanish or Haitian Creole had increased odds of positive Zika virus screening during pregnancy. These 2 findings are relevant to the design and implementation of public awareness campaigns.

The findings of our study are subject to several limitations. The high rate of loss to follow-up was due in part to screening only at delivery or late during pregnancy, resulting in discharge before receipt of results. Reengagement with this patient population has been difficult. Because our study was a retrospective chart review, we relied on accurate documentation of symptoms potentially attributable to Zika virus, which possibly limited detection of women who might have been symptomatic. Also, as tertiary care centers, we frequently receive patients who were initially managed at outlying clinics, and complete records were often fragmented, particularly in terms of PRNT results. In addition, because not all infants were tested for Zika virus at birth or were fully evaluated, we might not have accurately represented the impact of congenital Zika syndrome in this cohort. 

The strengths of this study include the large number of pregnant women screened for Zika virus in a diverse patient population. The wide socioeconomic strata represented by these women enabled identification of factors associated with the odds of screening positive for Zika virus during pregnancy.

Among the multiple patient management and counseling issues our study raises are the caveats in laboratory result interpretation and the need for initial counseling that provides the most current understanding of Zika virus infection during pregnancy. In addition, our study provides lessons for other regions at risk for local transmission. Specifically, the increase in screening for Zika virus can overwhelm hospital and public health systems, resulting in delayed receipt of results of screening and confirmatory tests. Similarly, delay in penetration of screening guidelines to the medical community may result in lack of screening during pregnancy, which can lead to missed cases or delayed diagnoses. Because the understanding of the effect of Zika virus infection during pregnancy and the guidelines regarding testing interpretation are rapidly evolving, clinicians need to be well-versed on the current national guidelines for Zika virus testing (*9**,**11*).
